# Beyond Symptom Relief: Quality of Life Recovery After Conservative Management of Early Hemorrhoids

**DOI:** 10.7759/cureus.106860

**Published:** 2026-04-11

**Authors:** Umang K Agrawal, Sakshi Jaiswal, Anand K Singh, Thrisha S Ajish, Antarik Sasmal, B M Navin, Ayush Jaiswal

**Affiliations:** 1 General Surgery, Institute of Medical Sciences, Banaras Hindu University, Varanasi, IND; 2 General Surgery, ESIC Medical College and Hospital, Varanasi, IND; 3 Surgery, Nalanda Medical College and Hospital, Patna, IND

**Keywords:** conservative management, haemorrhoids, haemorrhoid severity score, quality of life, retrospective study

## Abstract

Introduction

Hemorrhoidal disease is one of the most common anorectal disorders encountered in surgical practice and significantly affects patients’ daily activities and well-being. Conservative management remains the first-line treatment for early hemorrhoids (Grade I and II), focusing primarily on symptom relief. However, the impact of conservative therapy on health-related quality of life has not been extensively evaluated. The present study aimed to assess symptomatic improvement and quality-of-life outcomes following conservative management in patients with early hemorrhoidal disease.

Methods

A retrospective observational study was conducted in the Department of General Surgery at ESIC Medical College and Hospital, Varanasi, India. Medical records of patients diagnosed with Grade I or Grade II internal hemorrhoids and managed conservatively were reviewed. Patients with a minimum follow-up duration of six weeks were included in the study. Symptom severity was assessed using the Hemorrhoid Severity Score (HSS). Health-related quality of life was evaluated using the Short Form-12 (SF-12) questionnaire, generating the Physical Component Score (PCS) and Mental Component Score (MCS). Baseline and follow-up scores were compared using paired statistical analysis. Subgroup analysis was performed to evaluate differences in improvement between Grade I and Grade II hemorrhoids.

Results

A total of 50 patients were included in the final analysis. The mean age of the study population was 45.04 ± 12.48 years, with equal gender distribution comprising 25 (50%) males and 25 (50%) females. Grade II hemorrhoids were present in 29 (58%) patients, while 21 (42%) had Grade I disease.

Following conservative management, a significant reduction in symptom severity was observed. The mean Hemorrhoid Severity Score decreased from 10.71 ± 2.01 at baseline to 5.57 ± 2.39 at follow-up (p < 0.001), indicating substantial improvement. Similarly, the mean Physical Component Score improved from 39.31 ± 2.78 to 49.18 ± 3.26 (p < 0.001), reflecting better physical functioning, while the mean Mental Component Score improved from 40.99 ± 2.79 to 49.51 ± 3.72 (p < 0.001), indicating enhanced psychological well-being.

Large effect sizes were observed for improvements in symptom severity and quality-of-life measures. Subgroup analysis demonstrated comparable improvement between Grade I and Grade II hemorrhoids. Additionally, no significant correlation was identified between the magnitude of symptom improvement and changes in quality-of-life scores.

Conclusion

Conservative management of Grade I and II hemorrhoids leads to significant reduction in symptom severity and substantial improvement in both physical and mental aspects of quality of life. These findings reinforce the role of conservative therapy as an effective first-line treatment strategy in early hemorrhoidal disease.

## Introduction

Hemorrhoidal disease is among the most frequently encountered anorectal disorders in surgical practice and represents a significant source of morbidity in adults [1]. These vascular cushions play a role in continence, but pathological enlargement or inflammation leads to symptoms such as bleeding, discomfort, pruritus, and prolapse [2].

Internal hemorrhoids are classified based on prolapse severity. Early-stage disease (Grade I and II) is typically managed non-operatively, whereas advanced disease may require procedural or surgical intervention [3].

Non-operative management includes dietary modification, fiber supplementation, stool softeners, sitz baths, and topical agents. Fiber therapy reduces straining and improves stool consistency, thereby decreasing symptom recurrence [4]. Flavonoids have also demonstrated beneficial venotonic and anti-inflammatory effects [5].

While symptom resolution is often used as the primary outcome, hemorrhoidal disease can substantially affect quality of life, including physical activity and psychological well-being [6]. Therefore, patient-reported outcomes are increasingly emphasized.

Validated tools such as the Hemorrhoid Severity Score (HSS) and Short Form-12 (SF-12) enable objective assessment of symptom burden and quality of life [7,8]. However, limited studies have evaluated the effect of conservative therapy using both measures.

This study aimed to evaluate symptom improvement and quality-of-life outcomes following conservative management in patients with Grade I-II hemorrhoids.

## Materials and methods

This retrospective observational study was conducted in the Department of General Surgery at ESIC Medical College and Hospital, Varanasi, India. The medical records of patients presenting to the surgical outpatient department between November 2025 and February 2026 were reviewed. The study included adult patients (≥18 years) diagnosed with Grade I or Grade II internal hemorrhoids based on clinical evaluation and confirmation by proctoscopic examination, which remains the standard diagnostic modality for internal hemorrhoidal disease [9,10].

Patients who were managed conservatively and had a minimum follow-up duration of six weeks with complete baseline and follow-up clinical documentation were included in the study. Patients with advanced hemorrhoidal disease (Grade III or IV), thrombosed hemorrhoids, prior anorectal surgery, associated anorectal conditions such as anal fissure or fistula, inflammatory bowel disease, colorectal malignancy, or incomplete medical records were excluded to maintain homogeneity of the study population.

All included patients received standardized conservative treatment in accordance with current clinical recommendations for early hemorrhoidal disease [11]. The treatment protocol consisted of dietary advice emphasizing increased fiber intake, adequate oral fluid consumption, and lifestyle modification aimed at reducing straining during defecation. Pharmacological management included stool softeners, topical anti-hemorrhoidal agents, and a short course of oral flavonoids. Dietary fiber supplementation is known to improve stool consistency and reduce straining, thereby decreasing hemorrhoidal symptoms, while flavonoids have been shown to exert venotonic and anti-inflammatory effects on hemorrhoidal vasculature [4,5].

Data were extracted from outpatient records using a structured data collection format. Demographic variables, including age and sex, were recorded. Clinical variables included grade of hemorrhoids, baseline symptom severity, and follow-up symptom severity. Outcome measures included the Hemorrhoid Severity Score (HSS), Physical Component Score (PCS), and Mental Component Score (MCS).

Symptom severity was assessed using the Hemorrhoid Severity Score (HSS), a validated tool that quantifies symptom burden based on five domains: pain, bleeding, pruritus, soiling, and prolapse. Each symptom is graded according to frequency, and the cumulative score reflects overall disease severity, with higher scores indicating more severe symptoms [12].

Health-related quality of life was assessed using the Short Form-12 (SF-12) Health Survey, a widely validated instrument derived from the SF-36 questionnaire [8]. The SF-12 evaluates both physical and mental health domains and generates two summary measures: the Physical Component Score (PCS), reflecting physical functioning and activity limitations, and the Mental Component Score (MCS), reflecting psychological well-being, emotional health, and social functioning. Scores are standardized to a population mean of 50, with higher scores indicating better quality of life.

All outcome measures were recorded at baseline and at follow-up. Changes in HSS, PCS, and MCS were calculated to assess treatment response.

Data were entered into Microsoft Excel (Microsoft Corp., Redmond, WA) and analyzed using SPSS v. 25 (IBM Corp., Armonk, NY). Continuous variables were expressed as mean ± standard deviation, and categorical variables were expressed as frequency and percentage using the N (%) format. A paired t-test was used to compare baseline and follow-up scores within the same group. An independent t-test was used to compare differences between Grade I and Grade II hemorrhoids. Pearson correlation analysis was performed to assess the relationship between changes in symptom severity and quality-of-life scores. The effect size was calculated using Cohen’s d to determine the magnitude of the treatment effect. A p-value of less than 0.05 was considered statistically significant.

## Results

Baseline characteristics

A total of 50 patients were included in the final analysis. The mean age was 45.04 ± 12.48 years, with 25 (50%) males and 25 (50%) females. Grade II hemorrhoids were present in 29 (58%) patients, while 21 (42%) had Grade I disease (Table 1).

**Table 1:** Baseline Characteristics of the Study Population (N = 50)

Variable	Value
Age (mean ± SD)	45.04 ± 12.48
Median age	45.5
Age range	24–65
Male	25 (50%)
Female	25 (50%)
Grade I hemorrhoids	21 (42%)
Grade II hemorrhoids	29 (58%)

Symptom severity

The mean HSS at baseline was 10.71 ± 2.01, indicating moderate-to-severe symptom burden. Following conservative management, the mean HSS decreased significantly to 5.57 ± 2.39.

The mean reduction in HSS was 5.14 ± 1.14, demonstrating substantial improvement in symptom severity. Paired comparison confirmed this reduction to be statistically significant (p < 0.001). The comparison of baseline and follow-up HSS values is presented in Table 2.

**Table 2:** Comparison of Hemorrhoid Severity Score Before and After Treatment "-" indicates not applicable or not calculated.

Parameter	N	Mean ± SD	Median	Range	Mean Difference	95% CI	t value	p value
Baseline HSS	50	10.71 ± 2.01	10.9	7.3–14	-	-	-	-
Follow-up HSS	50	5.57 ± 2.39	5.45	0.9–9.8	5.14	4.81–5.46	31.91	<0.001

Physical quality of life

The mean PCS at baseline was 39.31 ± 2.78, indicating impaired physical quality of life. After conservative treatment, PCS improved significantly to 49.18 ± 3.26. The mean improvement in PCS was 9.87 ± 1.99, which was statistically significant (p < 0.001). The comparison of baseline and follow-up PCS values is shown in Table 3.

**Table 3:** Comparison of PCS Before and After Treatment "-" indicates not applicable or not calculated. PCS: Physical Component Score

Parameter	N	Mean ± SD	Median	Range	Mean Difference	95% CI	t value	p value
Baseline PCS	50	39.31 ± 2.78	39.75	34.3–43.6	-	-	-	-
Follow-up PCS	50	49.18 ± 3.26	49.15	40.7–56	9.87	9.30–10.43	-35.1	<0.001

**Figure 1:**
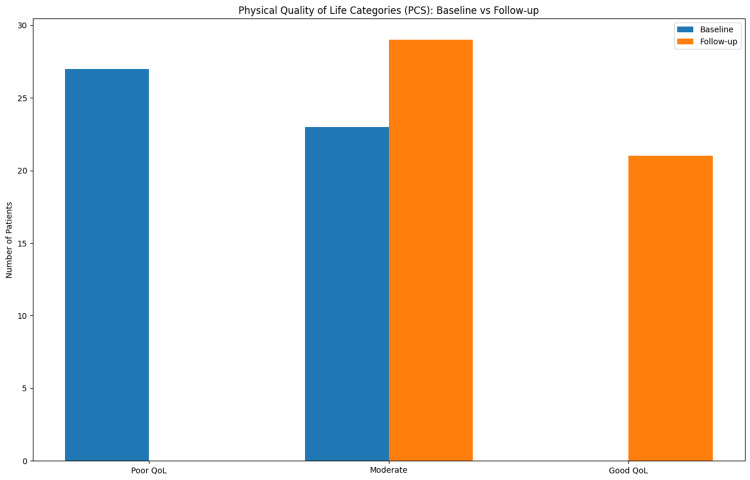
Distribution of Physical Quality-of-Life Categories Before and After Treatment. Bar chart showing the proportion of patients categorized as poor, moderate, and good quality of life based on PCS at baseline and follow-up. Blue bars represent baseline values and orange bars represent follow-up values. PCS: Physical Component Score

At baseline, 27 (54%) patients had poor quality of life, while 23 (46%) had moderate impairment. At follow-up, 29 (58%) patients had moderate impairment, and 21 (42%) achieved good quality of life (Figure 1).

Mental quality of life

The mean MCS at baseline was 40.99 ± 2.79, reflecting moderate impairment in psychological well-being. Following treatment, MCS improved to 49.51 ± 3.72, with a mean increase of 8.52 ± 2.36, which was statistically significant (p < 0.001). The comparison of baseline and follow-up MCS values is shown in Table 4.

**Table 4:** Comparison of the MCS Before and After Treatment. "-" indicates not applicable or not calculated. MCS: Mental Component Score

Parameter	N	Mean ± SD	Median	Range	Mean Difference	95% CI	t value	p value
Baseline MCS	50	40.99 ± 2.79	41.25	36.6–46	-	-	-	-
Follow-up MCS	50	49.51 ± 3.72	50.05	41.8–56.8	8.52	7.85–9.19	25.52	<0.001

**Figure 2:**
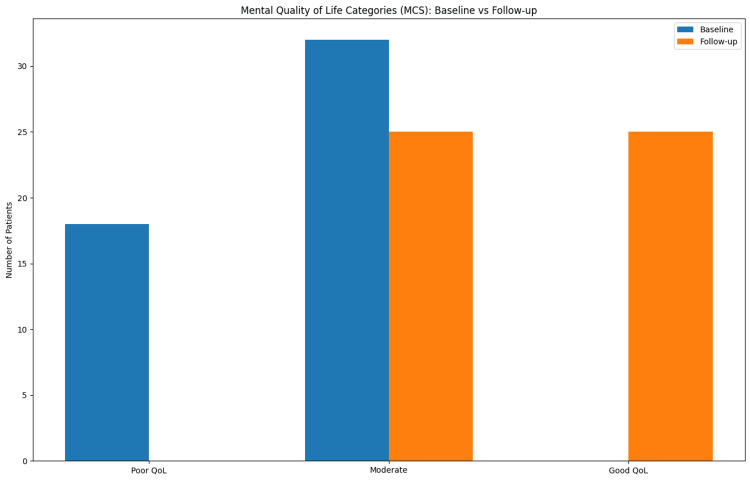
Distribution of Mental Quality-of-Life Categories Before and After Treatment. Bar chart illustrating changes in mental quality-of-life categories based on the MCS at baseline and follow-up. Blue bars represent baseline values and orange bars represent follow-up values. MCS: Mental Component Score

At baseline, 18 (36%) patients had poor quality of life, while 32 (64%) had moderate impairment. At follow-up, 25 (50%) patients had moderate impairment, and 25 (50%) achieved good quality of life (Figure 2).

Quality of life distribution 

Changes in mental and physical quality-of-life categories were observed following conservative treatment. This is summarized in Table 5.

**Table 5:** Quality of Life Distribution (PCS + MCS) QoL: quality of life; PCS: Physical Component Score; MCS: Mental Component Score

Category	PCS Baseline	PCS Follow-up	MCS Baseline	MCS Follow-up
Poor QoL	27 (54%)	0 (0%)	18 (36%)	0 (0%)
Moderate QoL	23 (46%)	29 (58%)	32 (64%)	25 (50%)
Good QoL	0 (0%)	21 (42%)	0 (0%)	25 (50%)

Grade-wise comparison of the improvement of symptoms and quality of life

The improvement in symptom severity and quality of life was comparable between Grade I and Grade II hemorrhoids. The mean reduction in HSS was 4.94 ± 1.11 for Grade I and 5.28 ± 1.15 for Grade II hemorrhoids (p = 0.30).

Similarly, PCS improvement: 10.13 ± 1.57 vs 9.68 ± 2.25 (p = 0.44), MCS improvement: 8.02 ± 2.31 vs 8.89 ± 2.37 (p = 0.20). These findings are summarized in Table 6.

**Table 6:** Grade-Wise Comparison of Improvement HSS: Hemorrhoid Severity Score; PCS: Physical Component Score; MCS: Mental Component Score

Outcome	Grade I (Mean ± SD)	Grade II (Mean ± SD)	p-value
ΔHSS	4.94 ± 1.11	5.28 ± 1.15	0.3
ΔPCS	10.13 ± 1.57	9.68 ± 2.25	0.44
ΔMCS	8.02 ± 2.31	8.89 ± 2.37	0.2

Correlation analysis and effect size

Correlation analysis demonstrated no statistically significant relationship between changes in symptom severity and quality-of-life scores (Table 7). Large effect sizes were observed for all outcomes, indicating a strong treatment effect (Table 8).

**Table 7:** Correlation Between Symptom Improvement and Quality of Life HSS: Hemorrhoid Severity Score; PCS: Physical Component Score; MCS: Mental Component Score

Variable	Pearson's r	p-value
ΔHSS vs ΔPCS	-0.026	0.86
ΔHSS vs ΔMCS	0.071	0.623

**Table 8:** Effect Size (Cohen’s d) HSS: Hemorrhoid Severity Score; PCS: Physical Component Score; MCS: Mental Component Score

Outcome	Effect Size (Cohen's d)
HSS improvement	2.32
PCS improvement	3.25
MCS improvement	2.59

## Discussion

Hemorrhoidal disease is a frequently encountered anorectal condition that can adversely impact patients’ daily activities and overall quality of life. Conservative management continues to be the preferred initial approach for early-stage hemorrhoids (Grade I and II). In the present study, we assessed the effectiveness of non-operative treatment in reducing symptom burden and improving health-related quality of life using validated instruments, namely the HSS and the SF-12.

Symptom improvement

The findings of this study demonstrate a marked reduction in symptom severity following conservative therapy. The mean HSS showed a significant decline from 10.71 ± 2.01 at baseline to 5.57 ± 2.39 at follow-up, indicating meaningful clinical improvement.

These observations are consistent with prior literature supporting the role of conservative measures in managing early hemorrhoidal disease. Increased dietary fiber intake has been shown to reduce bleeding and improve bowel habits by enhancing stool bulk and minimizing straining [13,14]. Alonso-Coello et al. further reported that fiber supplementation significantly lowers the likelihood of persistent symptoms in hemorrhoidal disease [4].

Pharmacological agents such as flavonoids are also believed to contribute to symptom relief through their venotonic and anti-inflammatory properties [5,13]. The substantial reduction in HSS observed in our study reinforces the effectiveness of conservative strategies in symptom control.

Improvement in the physical quality of life

Beyond symptom reduction, a significant improvement in physical functioning was observed. The mean PCS increased from 39.31 ± 2.78 to 49.18 ± 3.26, reflecting a notable enhancement in physical quality of life.

Symptoms such as bleeding, discomfort, and prolapse can limit physical activity and interfere with routine tasks. Improvement in PCS suggests that conservative therapy not only addresses symptomatology but also restores functional ability.

Comparable findings have been reported in studies evaluating patient-reported outcomes in anorectal disorders. Abramowitz et al. demonstrated that hemorrhoidal disease significantly impairs quality of life and that effective treatment leads to measurable improvement in physical functioning [6].

Improvement in the mental quality of life

The present study also demonstrated significant improvement in psychological well-being, as evidenced by an increase in mean MCS from 40.99 ± 2.79 to 49.51 ± 3.72.

Hemorrhoidal disease is often associated with psychological distress due to discomfort, embarrassment, and fear of recurrent symptoms. The observed improvement in MCS indicates that conservative management may also alleviate these emotional and social burdens.

There is growing recognition of the importance of mental health assessment in anorectal disorders. Previous studies have shown that treatment of hemorrhoidal disease results in improvements not only in physical symptoms but also in emotional well-being and social functioning [13,15].

Grade-wise comparison

No statistically significant difference was observed between Grade I and Grade II hemorrhoids in terms of improvement in symptom severity or quality-of-life scores. This suggests that conservative therapy provides comparable benefits across the early stages of hemorrhoidal disease.

These findings are in agreement with current clinical recommendations that advocate conservative management as the initial approach for both Grade I and Grade II hemorrhoids prior to considering procedural interventions [10].

Correlation between symptom improvement and quality of life

Interestingly, no significant association was identified between the degree of symptom improvement and changes in quality-of-life scores. This finding suggests that quality of life in hemorrhoidal disease is influenced by multiple factors beyond symptom severity alone.

Individual perception of health, psychological status, and social context may all contribute to overall quality-of-life outcomes. Similar findings have been reported in colorectal research, where clinical improvement does not always directly correspond to patient-perceived well-being [16].

Strengths

This study has several notable strengths. It utilized validated scoring systems to assess both symptom severity and quality of life. Additionally, the inclusion of equal numbers of male and female participants minimizes gender-related bias. The use of effect-size analysis further strengthens the findings by demonstrating a substantial treatment impact.

Limitations

Despite these strengths, certain limitations must be acknowledged. The retrospective design introduces the possibility of selection bias. The study was conducted at a single center with a relatively small sample size, which may limit generalizability. Furthermore, the follow-up duration was relatively short and may not fully reflect long-term outcomes.

Future prospective studies with larger cohorts and longer follow-up periods are warranted to further evaluate the sustained impact of conservative management on quality-of-life outcomes in hemorrhoidal disease.

## Conclusions

Conservative management is highly effective in reducing symptom severity and improving quality of life in patients with Grade I and Grade II hemorrhoids. Significant improvements were observed in Hemorrhoid Severity Score as well as in both physical and mental health domains, indicating meaningful recovery in functional and psychological well-being.

These findings support the continued use of conservative therapy as the first-line treatment for early hemorrhoidal disease. Given its effectiveness, safety, and accessibility, conservative management plays a crucial role in reducing disease burden and improving patient-centered outcomes.
